# Etiological Influences on Perceptions of Parenting: A Longitudinal, Multi-Informant Twin Study

**DOI:** 10.1007/s10964-016-0419-0

**Published:** 2016-01-27

**Authors:** Laurie J. Hannigan, Tom A. McAdams, Robert Plomin, Thalia C. Eley

**Affiliations:** Medical Research Council, Social, Genetic and Developmental Psychiatry Centre, Institute of Psychiatry, Psychology and Neuroscience, King’s College London, De Crespigny Park, Denmark Hill, London, SE5 8AF UK

**Keywords:** Parenting, Behavioral genetics, Environmental, Longitudinal, Child development, Adolescence

## Abstract

Children and their parents often differ in their perception of the relationship they share. As this relationship changes developmentally, the nature of these differences may also change. Longitudinal genetic designs can be used to investigate the developmental etiologies of shared and distinct perceptions. In this study, we used longitudinal psychometric models to analyze child and parent reports of negative parenting for 6417 twin pairs from the Twins Early Development Study at ages 9, 12 and 14 years. Within-time cross-reporter correlations, indicating the degree to which children and parents perceived negative parenting behaviors similarly at each age, were moderate (*r* = .44 − .46). Longitudinal genetic analyses revealed these shared perceptions to be relatively stable during the transition into adolescence, with this stability driven by a combination of children’s genetic factors and family-wide environmental factors. In contrast, child- and parent-specific perceptions of parenting were predominantly age-specific, a developmental pattern underpinned by child genetic factors and a combination of family-wide and unique environmental influences. These results and their implications are discussed in the context of interplay between reciprocal interactions, subjective insight and developmental behavioral change in the parent–child relationship.

## Introduction

The strategies adopted by parents in raising their children—as well as the manner in which these are employed—have long been viewed by theorists as being central to child development (Belsky 1984; Maccoby 1992). However, it is clear that the nature of the parent–child relationship is by no means dictated by just one member of the dyad (Bell 1968, 1979; Paschall and Mastergeorge 2015; Pettit and Arsiwalla 2008). Nor can parenting realistically be considered an objective, unified set of processes and interactions that child and parent necessarily experience similarly (De Los Reyes 2011; Taber 2010). Instead, a complexity arising from the interdependence of child and parent behaviors is further enhanced by the potential for differences in their respective experiences.

For researchers studying child development, unpicking this complexity represents both a methodological challenge and a potentially fruitful avenue for research. The parent–child relationship is, in itself, subject to developmental change. Given that the transitional period between childhood and adolescence is particularly salient in this regard (Eccles et al. 1993; Laursen and Collins 2009; Laursen et al. 1998; Paikoff and Brooks-Gunn 1991; Steinberg 2000), it represents an important opportunity to ask questions exploring the interplay between children and parents’ behavior and perceptions. For example: how distinct are children and parents’ perceptions of parenting during this period? Do they differ from one another to a greater degree as children progress into adolescence? What factors underpin the differences (and similarities) in how parenting is perceived? How do these factors cooperate to drive developmental stability, or change, in the parent–child relationship? Answering such questions will help to make sense of the nuances in the parent–child relationship, and to clarify how associations between parenting and developmental outcomes may evolve in adolescence.

### Perceptions of Parenting: Shared and Individual Reality

Observational techniques are widely seen as the “gold standard” for the empirical measurement of parenting (Smith 2011; e.g., Bradley et al. 1989). Studies using observational measures of parenting and have provided an array of valuable insights into how parents’ behavior is associated with developmental outcomes (Bradley et al. 2001a, b; Han et al. 2004). Nonetheless, the use of psychometric questionnaires to measure parenting is also widespread (Hurley et al. 2014; Locke and Prinz 2002). Often, this simply reflects pragmatic considerations, such as unfeasibility or prohibitive cost of observation when large sample sizes are sought. However, despite valid concerns over their objective reliability (Morsbach and Prinz 2006; Taber 2010), there are some potential empirical benefits in the use of questionnaire data. The nature of the parenting relationship is such that it only exists in the interactions between parent and child. Thus, it is not unreasonable to consider that the individuals involved may be uniquely positioned to inform about its functioning. Indeed, it has been suggested that there may exist a subjective, family reality, to which an outside observer has limited access (Feinberg et al. 2001). When studying the parenting of older children and adolescents, it becomes possible to consider the extent to which this family reality may coexist with separate—yet valid—individual realities, corresponding to the unique perspectives of children and parents (De Los Reyes 2011). This idea is supported by the conspicuously low level of agreement between child- and parent-reports of parenting (Bell et al. 2001; Feinberg et al. 2001; Reidler and Swenson 2012).

An individual’s subjective perception of the parenting they receive (or, in the case of parents, the parenting they provide) may be at least as important for family functioning and child development as any “objective” reality (Abar et al. 2015; Feinberg and Hetherington 2001; Maurizi et al. 2012). For example, an adolescent’s perception of their parent’s disciplinary behavior may relate to subsequent developmental outcomes, irrespective of how *objectively* harsh that behavior may be (Feinberg et al. 2000; Guion et al. 2009; Neiderhiser et al. 1998). Conceivably, the mechanisms driving these relationships may also differ depending on whether discipline is subjectively or objectively harsh (e.g., Gunlicks-Stoessel and Powers 2008). At the core of the parent–child relationship, the shifting delineations between family-level “shared reality” and individual-level “subjective reality” may have profound implications for its functioning and impact on the developing child (Carlson et al. 1991). Such a possibility has prompted some researchers to advocate an increased emphasis on reporters’ individual insights (Collins 1991; Feinberg et al. 2000). This calls for the investigation—rather than avoidance—of subjectivity; an approach used increasingly in questionnaire-based parenting research, including work on parental monitoring (Abar et al. 2015; de Los Reyes et al. 2010; Reynolds et al. 2011), influence (McElhaney et al. 2008), conflict-negativity (Guion et al. 2009; Neiderhiser et al. 1998), general parenting behavior and family functioning (Maurizi et al. 2012; Ohannessian and De Los Reyes 2014; Stuart and Jose 2012), and parent–child relationships in the context of divorce (Pelton and Forehand 2001) and maternal illness (Pelton et al. 2001). With increasing interest in this approach, some caution has been advised regarding the use of difference scores to index parent–child discrepancies, and specific methodological refinement called for in future work (Laird and De Los Reyes 2013). Nonetheless, the potential for meaningful contributions from this area of study has been well demonstrated.

### Gene-Environment Correlation in Studies of Parenting

Developing a clearer understanding of the nature of the overlap and differences between parent and child perspectives on parenting is important, particularly in the context of burgeoning interest in the functional consequences of cross-reporter discrepancies. Studies using genetically informative data have already begun to play an important role in this process, by clarifying the nature of the phenotypic information captured by measures of parenting. In standard quantitative genetic designs, questionnaire responses from/about twins or adopted siblings are analyzed to reveal the underlying etiological structure of traits and behaviors. With parenting often characterized as a source of environmental risk for child and adolescent development, the application of a quantitative genetic design to a parenting phenotype allows researchers to quantify the extent to which its measurement is confounded by *gene*-*environment correlation*—whereby an individual’s environmental exposure is associated with their genotype (Plomin and Bergeman 1991). This can occur via different mechanisms stemming from the interrelationship of genetic and environmental factors in a home shared by biologically related family members (Plomin et al. 1977; Scarr and McCartney 1983). Genetic influence on measures of the environment that is mediated via others’ responses to an individual’s genetically-influenced behavior is known as *evocative* gene-environment correlation. For example, measures of parental control may be confounded by evocative gene-environment correlation if children’s genetically influenced behavior influences the extent to which their parents feel the need to regulate their activities. Where parents’ own genes influence their parenting behaviors, *passive* gene-environment correlation may also come into effect, as children inherit some of these genes, which are thus associated with the parenting they experience (Neiderhiser et al. 2004; Plomin et al. 1977). A third type, *active* gene-environment correlation, refers to associations driven by individuals’ tendencies to seek out environments suited to their genetic predispositions. Active gene-environment correlation is usually considered as more likely in regard to environmental factors outside of the parent–child relationship (Neiderhiser et al. 2004).

Genetically sensitive analyses of parenting data have routinely found evidence of gene-environment correlation (see reviews by Kendler and Baker 2007; Klahr and Burt 2014; McAdams et al. 2014; Plomin et al. 1994). The forms of gene-environment correlation detectable in such analyses depend upon the nature of the data being studied, and on aspects of the study design. For example, for the detection of passive gene-environment correlation in relation to parenting, parent-based studies (such as the children-of-twins design) are most suitable. In contrast, child-based designs, such as the classical twin study, can more effectively isolate the effects of evocative/active gene-environment correlation when parenting is the phenotype under study (Neiderhiser et al. 2004). While the evocative and active forms of gene-environment correlation are difficult to separate empirically, there is a strong theoretical basis for the usual emphasis on the former in the interpretation of results from studies of parenting (i.e., the relative plausibility of children evoking responses from their parents *versus* selecting different parenting environments to which they will be exposed). Numerous studies have demonstrated the key role evocative gene-environment correlation plays in shaping the parent–child relationship (e.g., Elkins et al. 1997; Ludeke et al. 2013; Oliver et al. 2013; Wade and Kendler 2000; Hannigan et al. 2015; meta-analysis by Avinun and Knafo 2014). These findings provide consistent empirical support for theoretical models that emphasize the importance of child-driven effects the parent–child relationship (e.g., Bell 1979; Cook and Kenny 2005).

### The Use of Genetic Data to Investigate Shared and Distinct Perceptions

Genetic studies incorporating data from multiple reporters can be used to address the issues of gene-environment correlation and subjectivity of experience in parallel. This is done by exploring the etiological underpinnings of different reporters’ shared and distinct perceptions of a phenotype simultaneously. Variance in a phenotype that is shared between reporters can be distinguished from variance that is unique to an individual’s report, before both (shared and unique variance) are decomposed into their genetic and environmental components. The structural equation models used to do this were referred to as *psychometric models* in the earliest instances of their use (Bartels et al. 2003b; Hewitt et al. 1992) and we retain this terminology throughout the current paper. These models can approximate some form of objectivity in extracting variance upon which reporters agree. Crucially though, this is done without either discarding or rendering uninterpretable any additional insights from individual reporters (Bartels et al. 2007a). The estimation of shared perceptions allows genetic influence on the target behavior, which *can* feature as such in the shared section of the models, to be separated from genetic influence on an individual’s perceptions or reporting biases, which cannot. The estimation of distinct perceptions allows the validity of the subjective insights of different reporters to be affirmed, as patterns of genetic influence in variance unique to one reporter cannot be mimicked by bias or error (Hoekstra et al. 2008). Psychometric models have been successfully applied to a range of specific child and adolescent behaviors, including: aggression (Bartels et al. 2003b; Hudziak et al. 2003; Tackett et al. 2009); antisocial behavior (Arseneault et al. 2003); anxiety (Ask et al. 2014); and obsessive compulsive behavior (van Grootheest et al. 2007).

**Fig. 1:**
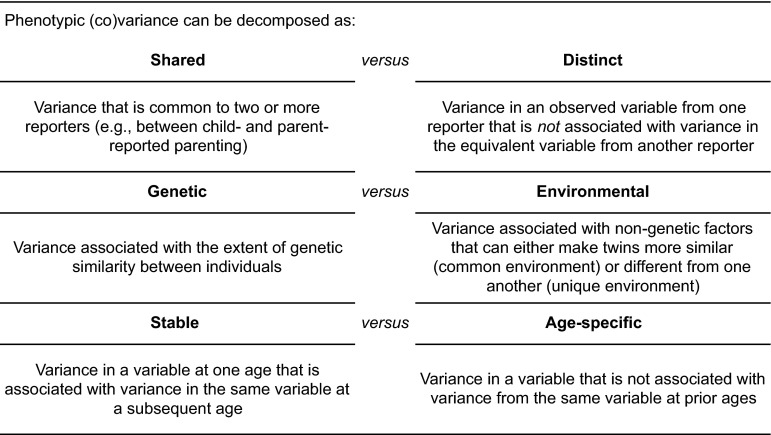
Decomposing variance in longitudinal psychometric models

The suitability of psychometric models for exploring developmental questions is further highlighted by the fact that they can also be extended for the analysis of longitudinal data (e.g., Bartels et al. 2007b; Hoekstra et al. 2008; Hudziak et al. 2003). Standard longitudinal genetic analyses allow for the estimation of the relative contributions of genetic and environmental factors to stability and change in a phenotype over time. This means that the etiology of the phenotype can be viewed in a developmental context, allowing more nuanced questions to be addressed (e.g., Do the same genes influence the trait of interest at different ages? Do environmental influences operate stably across time?). In the longitudinal extension of the psychometric model, variance in a phenotype can thus be sub-divided at three levels: shared versus distinct; genetic versus environmental; and stable versus age-specific (see Fig. 1). The benefits of this are particularly relevant for the study of the parent–child relationship across development, where the interplay between age-related change and trait- or environmentally-mediated stability is evident. By examining results in different sections in the models with reference to expectations about the longitudinal effects of wider environmental factors, reporting biases and measurement error, the influence of child and parent behaviors on the parenting relationship can be inferred.

Previous examples of multiple-reporter, genetically informative studies of parenting are limited. One early study using a multiple-informant, child-based, genetic design looked explicitly at the mediating role of adolescent perceptions of parenting on the relationship between parental conflict and negativity. Adolescents’ perceptions of parenting were found to mediate this association, with genetic factors implicated in the underlying etiological overlap between the phenotypes (Neiderhiser et al. 1998). A subsequent study on the same dataset analyzed parenting reports from children, parents and observers to investigate genetic and environmental influences at three levels: variance common to all three reporters was interpreted as “social reality”; variance shared between children and parents as a “family reality” and variance specific to parent reports as “individual reality”. Genetic influences, which can be interpreted as the effects of child behavior on parenting (i.e., evocative gene-environment correlation) in child-based studies, were significant at the social and family levels, whereas parent-specific variance was largely subject to common environmental influences (Feinberg et al. 2001). A further study explicitly compared the etiological architecture of adolescent-, mother- and observer-reports of mothers’ parenting behaviors in two samples (Neiderhiser et al. 2004). While observer reports of maternal negativity and control were influenced only by environmental factors, genetic factors did influence both mother- and adolescent-reports. However, the extent to which these etiological factors overlapped across reporters was not determined. To our knowledge, no previous study has examined the developmental etiology of adolescents’ and parents’ shared and distinct perceptions of parenting using longitudinal psychometric models.

## Overview and Hypotheses

In the current study, we took a developmental approach to examining the etiologies of the shared and distinct perceptions of parenting held by children and their parents, during the transition into adolescence. We did this by applying longitudinal, psychometric structural equation models to child and parent reports of parenting in a genetically informative dataset. Specifically, ours is a child-based^1^ genetic design, in which genetic influence on the phenotype pertains to the role of child genes, irrespective of reporter.

Investigating stability and change in the etiologies of shared and distinct perceptions of parenting has several key implications for improving future research in this area. First, the importance of child-driven effects on parenting practices across development has been widely acknowledged and demonstrated (Avinun and Knafo 2014; Cook and Kenny 2005; Paschall and Mastergeorge 2015; Pettit and Arsiwalla 2008). The current study is designed to investigate the nuances in these effects, both in terms of age-specificity (do genetically-influenced child characteristics influence parenting consistently, or do they change as the child enters adolescence?) and in terms of perceptions (do children and parents perceive all child-driven effects on parenting commonly, or is either party unaware of some of the ways in which children evoke parenting responses?).

Second, the parent–child relationship is subject to substantial changes during the child’s transition to adolescence (Eccles et al. 1993; Laursen and Collins 2009; Paikoff and Brooks-Gunn 1991). This study seeks to ascertain to what extent children and parents perceive the changing aspects of their relationship jointly *versus* distinctly. This can inform as to whether or not perceptual discrepancies between parent and child can be a considered a potential mechanism by developmental changes in the parent–child relationship are mediated.

Third, there is growing interest in how investigations of subjectivity in the home environment can contribute (alongside more objective measures) to our understanding of child development (e.g., Abar et al. 2015; De Los Reyes 2011; Ohannessian and De Los Reyes 2014). The findings from the current study will help to clarify the functional consequences of parent–child discrepancies, in terms of the etiological nature and developmental structure of each reporter’s distinct perspective, and how these differ from the perspective they share.

Our two primary aims and relevant alternative hypotheses are outlined below. Of note, we refer primarily to *child*-reports and *children’s* perspectives throughout the description of the methodology and results in this report, irrespective of the age of the individuals in question (i.e., as opposed to *adolescent*). We do this to maintain clarity in respect of the two types of reporter in the study: parents and their children. We resume the use of developmentally-appropriate terminology (i.e., *adolescents*), where possible, in the discussion.

Our main aim was to investigate the sources of genetic influence on reports of parenting by comparing the contributions of genetic factors to shared, child-specific and parent-specific perceptions of parenting across development. We expected variance in both reporters’ reports of parenting to be subject to genetic influence, in line with previous work. If children’s genetically influenced behavior affected parenting in ways that both reporters perceived similarly, we expected to see genetic influence on shared variance, indicating the presence of evocative gene-environment correlation. Conversely, to the extent that both reporters perceived different effects of child behavior on parenting, we would see genetic influence on variance that was distinct to each reporter. In the case of parent-specific perceptions, this would be similarly indicative of evocative gene-environment correlation for parenting responses, but would pertain specifically to child-driven effects that the children themselves did not perceive. In the case of child-specific perceptions, genetic influence could result from a reversal of this scenario (i.e., evocative effects on parenting perceived exclusively by children), but also from children’s genetically-influenced perceptions or reporting tendencies. Finally, if the child behaviors influencing parenting were different at each age, we expected to see new genetic influences emerging at each wave. Conversely, if stable, trait-like child behaviors were involved, we would see evidence of genetic stability across time.

Our secondary aim was to compare the environmental components of shared and distinct perceptions of parenting, with reference to their stability (or instability) over the course of the transition into adolescence. Where reports of parenting indexed processes that were independent of children’s genetically influenced behavioral characteristics, we expected to see environmental influence on our variables. Furthermore, to the extent that these were “true” environmental influences—rather than the result of bias and/or measurement error—we anticipated that they would be jointly perceived and, thus, associated with shared variance between children and parents. Finally, if these aspects of parenting were stable over time, we expected to see environmental influences from early waves continuing to explain variance across development. In combination, these two broad aims and associated alternative hypotheses constitute an investigation of the shared and individual realities of the parent–child relationship between late childhood and mid-adolescence.

## Methods

### Participants

The study sample consisted of parents and twins enrolled in the on-going, longitudinal Twins Early Development Study (TEDS; Haworth et al. 2013). TEDS is a population-based twin sample consisting, at its inception, of all twins born in England and Wales between 1994 and 1996. Respondent families at first contact (N = 13,694) were highly representative of the UK population in terms of ethnicity and socio-economic status as well as gender and zygosity of twins (Haworth et al. 2013). As with most longitudinal studies, the number of families actively participating in the study has reduced over time for various reasons (e.g. active withdrawal, non-response to mailings, address problems, novel medical exclusions). Attrition in the TEDS sample as a whole is statistically associated with male gender, DZ zygosity and non-white ethnicity. In addition, the mean socio-economic status (SES) of the TEDS sample has gradually increased over time (standardized SES scores 0.13/0.19/0.24 for the 9-, 12-, and 14-year waves used in the current study). Nonetheless, the TEDS sample remains broadly representative of the UK population (Haworth et al. 2013).

Zygosity of twins in the TEDS sample was determined by parent ratings on questionnaire measures of twin similarity. Collection of DNA from more than 7000 TEDS twins has allowed for the validation of parent-ratings of zygosity, with results indicating an accuracy rate of >95 % (Price et al. 2000). Opposite-sex DZ pairs were included in the analyses. A scalar was included for all male variance parameters to account for sex differences in variance. This means that, while the proportion of variance attributable to genetic and environmental parameters in the models was constrained to be equal for males and females, the overall magnitude of variance was allowed to differ between the sexes. The significance of the scalar (and, thereby, the presence of such sex differences in the data) was tested during the model-fitting process.

TEDS participants included in the current study (N = 12,834 individual twins) were those children for whom completed measures of parenting, administered via postal questionnaire, were returned from at least one of three waves of data collection: ages 9, 12 and 14 years. Parent-reports came overwhelmingly from biological mothers of the twins (98.6 % as ascertained at age 9). The sample was 53 % female overall (55 % within MZ twins), and 93 % were of white European ethnicity. Sample sizes (presented by zygosity with descriptive statistics in the results section) varied by wave and variable. One notable feature of the TEDS study design is that it is divided into three cohorts (based upon year of birth). At some waves only two of these cohorts were approached to provide data. This explains the increase in sample sizes, in this study, between the 9-year wave (two cohorts) and the 12-year wave (three cohorts). 3340 individuals provided data at all waves, with a further 2064 individuals providing data at both three-cohort waves (12 and 14). Overall, 6 % of the current study sample was subject to exclusion for either medical or practical reasons (e.g., lack of zygosity information).

### Measures

#### Negative Parenting

Overall levels of negative parenting were indexed by a composite of items from two initial sets, designed to assess a range of parenting behaviors. The first assessed aspects of parental discipline via parent and child responses on six items derived from the parenting domain of a semi-structured interview (see Deater-Deckard et al. 1998). Participants reported how often parents’ used various disciplinary strategies (smacking/slapping; telling off/shouting; explaining/reasoning; being firm/calm; making a joke; and asking someone else to help) to deal with instances of child misbehavior.

The second set of items was designed to assess the affective domain of the parent–child relationship. Three positive items—“I feel happy about my relationship with my child”; “I am amused by my child”; “I feel close to my child” (wordings from parent-report version)—were included, alongside four negative items: “My Mum/Dad gets impatient with me”; “My Mum/Dad sometimes wishes I would leave him/her alone for a few minutes”; “I make my Mum/Dad angry”; “I make my Mum/Dad feel frustrated” (wordings from child-report version). These items were originally drawn from the Parental Feelings Questionnaire (PFQ; Deater-Deckard and O’Connor 2000).

Responses to all items were given on a 3-point scale, with the options: “Rarely/never”; “Sometimes”; and “Often” in the parent version, and “Not true”; “Quite true”; and “Very true” in the child version. All positive items (including those, from the first set, pertaining to calm or constructive parenting behaviors) were reverse-scored, such that higher scores overall would reflect more negative parenting.

With a view to maximizing reliability for the genetic analyses, we created a composite score from these items. To identify which items should be included in our composite indexing generally negative parenting, we performed exploratory factor analyses of all items for each reporter (child/parent) at each measurement occasion (9/12/14 years). One predominant factor was evident in each case, with eigenvalues >2 (and no other single factor eigenvalues >1). On examining the item loadings for this factor at each measurement occasion, we observed that the same four items loaded least (typically <0.3) on a majority of occasions and across reporters. The face validity of these four items (explaining/reasoning; making a joke; asking someone else to deal with the situation; parent amused by child) was reviewed and they were duly dropped, while the remaining nine items were combined to create the composite. On average, the internal consistency for this composite scale was acceptable for both parent report (Cronbach’s *α* range 0.71–0.72) and child report (Cronbach’s α range 0.70–0.76).

Parents gave their responses to each item for the elder and younger twin in turn. This mode of distinguishing twins from one another, as well as the order in which twins were referred to, was used consistently throughout questionnaires at each wave (and across TEDS as a whole). In addition, parents were first asked to identify, by name, which twin was the elder and which was the younger, in order to minimize the chance of confusion. In the child-report versions, twins reported only on their own experience.

### Statistical Analyses

#### Data Preparation

Raw data were regressed on age and sex in order to prevent the artificial inflation of twin correlations (McGue and Bouchard 1984). The distributions of scores on the composite parenting scale approximated normality for both raters. Accordingly, untransformed, standardized residuals of the age- and sex-regressed raw scores were used in the analyses.

#### Classical Twin Design

The models used in this study are grounded in the classical twin design. This design compares the phenotypic similarity of MZ twins, who share 100 % of their genes, with that of DZ twins, who share on average 50 % of their segregating genetic material. In contrast, MZ and DZ twins share their rearing environment to the same extent. This combination of systematic differences in the genetic relatedness of MZ *versus* DZ twin pairs and similarity in the extent of covariance in either type of twins’ rearing environments allows the sources of phenotypic variance to be ascertained. To the extent that MZ twins are more similar than DZ twins, additive genetic factors (A) are implicated. Any remaining phenotypic similarity that is evident to the same extent in both types of twins (i.e., DZ twin correlations are more than half the magnitude of MZ twin correlations) is attributable to the common environment (C). Finally, any influences that make MZ twins (who have correlations of 1 for both A *and* C) in the same family different from one another are accounted for by unique environmental factors (E).

#### Genetic Analyses

**Fig. 2:**
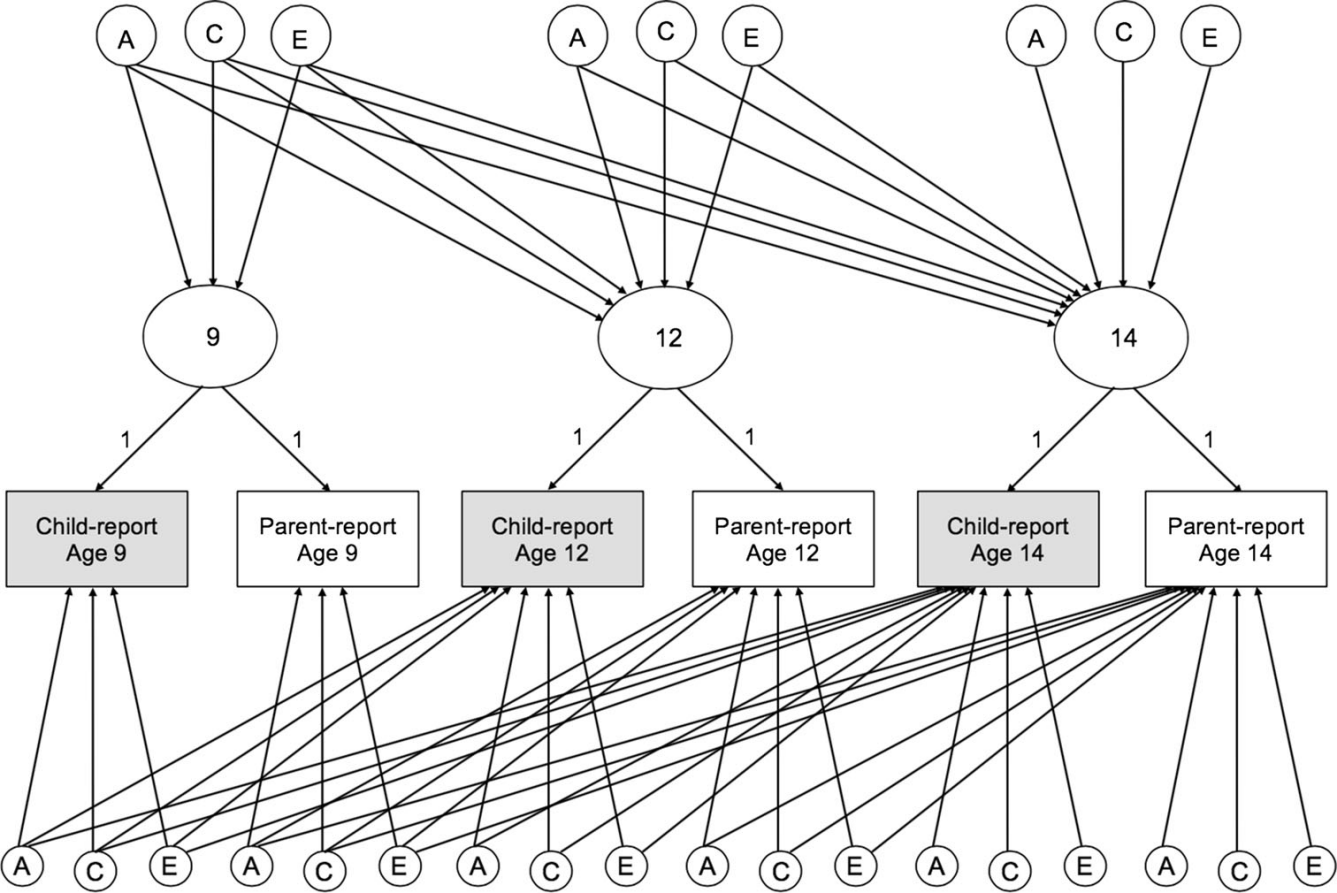
Longitudinal psychometric model for six observed variables (from two reporters) across 3 waves of data collection. *Note*: A = Additive genetic; C = Common environmental; E = Unique environmental; Shared perceptions indexed by latent factors in top half, distinct perceptions by residual variance below; the model shown is for one member of a twin pair only

In order to separately decompose the shared and distinct variance in child- and parent-reported parenting data, we fitted psychometric models (Bartels et al. 2003a; Hewitt et al. 1992; Hoekstra et al. 2008). The specification of the model as used in this study is shown, for one member of a twin pair, in Fig. 2. In the upper part of the model, latent factors indexing the within-age agreement between reporters are decomposed into additive genetic, common environmental and unique environmental components of variance. As well as accounting for a portion of the variance at their age of origin, these variance components can also explain variance in latent agreement factors at later ages. This occurs via the diagonal A, C and E paths. In this way, the upper section of the model allows the estimation of the importance of etiological influences from age 9 on shared perceptions of parenting at age 12 and 14 (and from age 12 on 14). Any variance that is unexplained by influences from earlier waves is decomposed into novel A, C and E components. The lower section of the model decomposes the residual variance in child- and parent-reports after their agreement is accounted for. Any perception held by an individual and not shared with their co-reporter will appear in this lower section of the model, alongside any measurement error and reporter-specific biases. These child- and parent-specific residuals are then decomposed into genetic, common environmental and unique environmental components. As in the upper part of the model, components of variance from earlier waves are able to account for (within-reporter) variance at later waves.

Model fitting was carried out in R using OpenMx (Neale et al. 2015). OpenMx uses full-information maximum likelihood (FIML) to estimate structural equation model parameters from transformed raw data, which reduces the impact of any bias from selective attrition in the sample (Enders and Bandalos 2001). FIML makes use of all available information in the raw data and is thus particularly efficient in dealing with missing data in general (Boker et al. 2015; Jelicić et al. 2009).

## Results

### Summary Statistics

Summary statistics, including analytic sample sizes by zygosity group, for raw scores on the negative parenting composite are presented in Table 1 by age and reporter. Small decreases in mean levels of negative parenting with increasing age were significant for both child-reports [*F* (2, 7803) = 331.26, *p* < 0.001] and parent reports [*F* (2, 7506) = 251.41, *p* < 0.001].

**Table 1:** Descriptive statistics for child- and parent-report raw scores on composite scale indexing levels of negative parenting

	Age	Mean	*SD*	N (MZ)	N (DZ)
Child	9	6.40	2.91	2178	3692
	12	5.59	3.04	3959	6947
	14	5.25	3.15	2358	3864
Parent	9	5.16	2.51	2360	4017
	12	4.65	2.51	3984	6986
	14	4.47	2.58	2269	3656

To test for the presence of selective attrition on the sample, we compared the age 9 raw scores of those individuals who contributed data throughout the study to those of individuals for whom data was unavailable at age 14. The results of these comparisons are presented in Table 2. Individuals providing data at all waves had marginally lower levels of negative parenting at age 9. This difference was just significant for both child-reports [*t* (5410) = −2.01, *p* < 0.05, *d* = 0.05] and parent-reports [*t* (5674) = −2.12, *p* < 0.05, *d* = 0.05], though effect sizes were small.

**Table 2:** Information on selective attrition in the study sample (scores on child- and parent-reported negative parenting composite scale)

	Mean (raw) score at age 9		*df*	t	*p*
	Data available at ages 9 & 14	Data unavailable at age 14			
Child	6.334	6.488	5409.5	−2.005	0.045
Parent	5.102	5.237	5674.1	−2.109	0.035

### Phenotypic Correlations

Within- and cross-reporter phenotypic correlations are reported in Table 3. Within-reporter correlations index the phenotypic stability of individuals’ reports of negative parenting over time. These correlations are generally moderate, ranging from 0.34 (child-reported parenting at age 9 and age 14) to 0.62 (parent-reported parenting age 12 and age 14). Phenotypic stability was, on average, slightly higher for parents’ reports. Within-age, cross-reporter correlations (presented on the diagonal of the cross-reporter section of the table) index the extent of the “agreement” between parents and their children on levels of negative parenting at each age. These correlations are also moderate, and are very similar at each measurement occasion, ranging from 0.44 to 0.46. These correlations indicate the amount of shared variance that will be decomposed in the upper part of the model presented in Fig. 2. Cross-reporter correlations presented above and below the diagonal index the across-time agreement between reporters. These correlations, indicating the overall stability of shared variance in scores on the negative parenting composite over time, range from 0.29 (for parent-reported parenting at age 9 and child-reported parenting at age 14) to 0.35 (for parent-reported parenting at age 12 and child-reported parenting at age 12).

**Table 3:** Within- and cross-reporter phenotypic correlations for child-and parent-reported negative parenting

	Within-reporter: child		Within-reporter: parent		Cross-reporter		
Age	9	12	9	12	9	12	14
9					0.45	0.33	0.29
12	0.41		0.61		0.33	0.44	0.35
14	0.34	0.49	0.54	0.62	0.31	0.34	0.46

Cross-reporter correlations: values below diagonal are for child report at age specified in row and parent report at age specified in column; values above the diagonal are for the reverse

### Twin Correlations

Within- and cross-reporter twin correlations are presented in Table 4. Notably, both MZ and DZ twin correlations were consistently higher for parent-reports; for example, at age 9, 0.93/0.71 (MZss/DZss; parent-report) versus 0.64/0.54 (child-report). In part, this may indicate that parents report parenting their offspring more similarly than offspring perceive. In addition, it may also reflect to the fact that, for parent-reports, the same individual reported on parenting behaviors for each twin, whereas for child-reports, each twin reported on their own parenting. Indeed, high correlations were observed for parent-reports of negative parenting throughout (e.g., for MZ twins: age 9: 0.93; age 12: 0.91; age 14: 0.92). DZ correlations are consistently lower than MZ correlations, indicating modest genetic influence on both child- and parent-reports of parenting. This is suggestive of some role for evocative gene-environment correlation in parenting, as expected. However, differences between MZ and DZ twin correlations are smaller than the difference in the genetic relatedness of the two twin types (MZ twins share 100 % segregating genes, DZ twins 50 %). This indicates that some non-genetic (i.e., common environmental) factors were operating to increase correlations between twins of both zygosities. The cross-reporter twin correlations are informative as to the likely characteristics of the decomposition of shared variance in the genetic modeling. The overall pattern of considerable similarity in MZ and DZ cross-reporter twin correlations suggests a prominent role for common environmental influences in explaining shared variance.

**Table 4:** Twin correlations within- and across- age and reporter

	Child			Parent			Cross-reporter		
Age	9	12	14	9	12	14	9	12	14
MZ (same sex)									
9	0.64			0.93			0.48		
12	0.39	0.58		0.59	0.91		0.33	0.44	
14	0.37	0.41	0.57	0.57	0.60	0.92	0.34	0.36	0.44
DZ (same sex)									
9	0.54			0.71			0.38		
12	0.35	0.43		0.45	0.74		0.29	0.33	
14	0.26	0.27	0.42	0.38	0.44	0.70	0.25	0.23	0.31
DZ (opposite sex)									
9	0.38			0.65			0.32		
12	0.23	0.40		0.45	0.72		0.29	0.34	
14	0.20	0.28	0.32	0.40	0.46	0.66	0.24	0.25	0.28

### Genetic Analyses

The longitudinal psychometric models used in the genetic analyses partitioned shared and distinct variance for decomposition, as well as estimating the influences of temporally distinct factors from within these sources. Fit statistics from the full psychometric model and sub-models testing the significance of specific parameters are presented in Table 5.

**Table 5:** Comparative fit statistics from model-fitting procedure

	ep	−2LL	*df*	AIC	ΔfLL	Δ*df*	*p*
Full psychometric model	66	105,881.4	46,204	13,473.4	NA	NA	NA
1. Drop scalar	60	105,920.3	46,210	13,500.3	38.91	6	0.00
2. Drop all NS paths	46	106,063.8	46,224	13,615.8	182.46	20	0.00

*ep* estimated parameters, *LL* log likelihood, *AIC* Akaike’s information criterion, *df* degrees of freedom; reduced models compared to full model

A comparison of the fit statistics for the full model and sub-model 1 confirmed the significance of the scalar for sex differences in variance (where its removal resulted in a significant decrement in fit). The next comparison shows the reduction in fit that resulted from dropping all paths that were non-significant in the full model. In large multivariate models, each dropped path may change the weighting of multiple remaining paths. As a result, the order in which paths are dropped can influence the nature of the reduced model that is ultimately seen to fit the data best. In the absence of an explicit theoretical basis for dropping paths in a particular order, we tested only this unbiased sub-model (2), which fit the data significantly worse than the full psychometric model. We therefore present and discuss the results of the full model, due to its inclusion of all non-significant paths, rather than what could only have been an arbitrary subset in any intermediately reduced sub-model.

Results from the full psychometric model are presented and described below in terms of relative genetic, common environmental and unique environmental factors explaining stability and change in, respectively, the shared and distinct perceptions of negative parenting held by children and their parents.

#### Shared Perceptions

Parameter estimates from the upper section of the model, which decomposes variance shared by both reporters (as shown in the top half of Fig. 2 and described in the methods section), are presented, with 95 % confidence intervals, in the first three columns of Table 6. Values in the first column index the proportionate influence of age 9 etiological factors on variance at each age. Values in the second column index the proportionate influence of age 12 etiological factors on variance at 12 and 14. Finally, values in the third column index the proportionate influence of age 14 etiological factors, which can explain variance at age 14 only. The overall proportion of phenotypic variance at each age that is explained by each set of factors is given in the “Total” rows. Totals for shared perceptions were similar to those initially indicated by the cross-reporter phenotypic correlations: 45 % at age 9; 43 % (0.27 + 0.16) at age 12; 46 % (0.22 + 0.08 + 0.16) at age 14.

**Table 6:** Genetic, common environmental and unique environmental contributions to shared and distinct perceptions of parenting derived from the full psychometric model of child- and parent-reported negative parenting at 9, 12 and 14 years

	Shared perceptions			Distinct perceptions					
				Child-specific			Parent-specific		
	Age 9 factors	Age 12 factors	Age 14 factors	Age 9 factors	Age 12 factors	Age 14 factors	Age 9 factors	Age 12 factors	Age 14 factors
9									
A	**0.18** *(.14*–*.22)*			**0.14** *(.06*–*.21)*			**0.34** *(.28*–*.37)*		
C	**0.26** *(.22*–*.31)*			0.04 *(.00*–*.10)*			**0.15** *(.10*–*.20)*		
E	0.01 *(.00*–*.02)*			**0.37** *(.34*–*.40)*			**0.06** *(.05*–*.08)*		
Total	**0.45**			**0.55**			**0.55**		
12									
A	**0.06** *(.03*–*.10)*	**0.11** *(.07*–*.14)*		0.04 *(.00*–*.14)*	0.11 *(.00*–*.17)*		**0.08** *(.05*–*.11)*	**0.16** *(.12*–*.20)*	
C	**0.21** *(.16*–*.26)*	0.03 *(.00*–*.08)*		0.02 *(.00*–*.05)*	0.00 *(.00*–*.05)*		**0.03** *(.01*–*.06)*	**0.23** *(.19*–*.27)*	
E	0.00 *(.00*–*.02)*	0.02 *(.00*–*.03)*		0.00 *(.00*–*.01)*	0.40 *(.38*–*.43)*		0.01 *(.00*–*.01)*	**0.06** *(.05*–*.07)*	
Total	**0.27**	**0.16**		**0.06**	**0.51**		**0.13**	**0.44**	
14									
A	**0.12** *(.07*–*.19)*	**0.04** *(.01*–*.09)*	**0.10** *(.05*–*.15)*	0.01 *(.00*–*.10)*	0.04 *(.00*–*.16)*	0.07 *(.00*–*.14)*	**0.05** *(.02*–*.08)*	**0.03** *(.01*–*.07)*	**0.15** *(.11*–*.20)*
C	**0.10** *(.06*–*.15)*	0.03 *(.00*–*.11)*	0.03 *(.00*–*.07)*	0.00 *(.00*–*.06)*	0.02 *(.00*–*.09)*	0.00 *(.00*–*.09)*	**0.06** *(.03*–*.08)*	**0.03** *(.02*–*.06)*	**0.18** *(.12*–*.22)*
E	0.00 *(.00*–*.03)*	0.01 *(.00*–*.04)*	0.03 *(.00*–*.04)*	0.00 *(.00*–*.01)*	0.01 *(.00*–*.02)*	0.39 *(.36*–*.43)*	0.00 *(.00*–*.01)*	0.00 *(.00*–*.00)*	**0.05** *(.04*–*.07)*
Total	**0.22**	**0.08**	**0.16**	**0.01**	**0.07**	**0.46**	**0.11**	**0.06**	**0.37**

A = genetic influences; C = common environmental influences; E = unique environmental influences; values are standardised, squared path estimates; significant estimates and totals in bold typeface; 95 % confidence intervals below point estimates in italics; results from full psychometric model

**Fig. 3:**
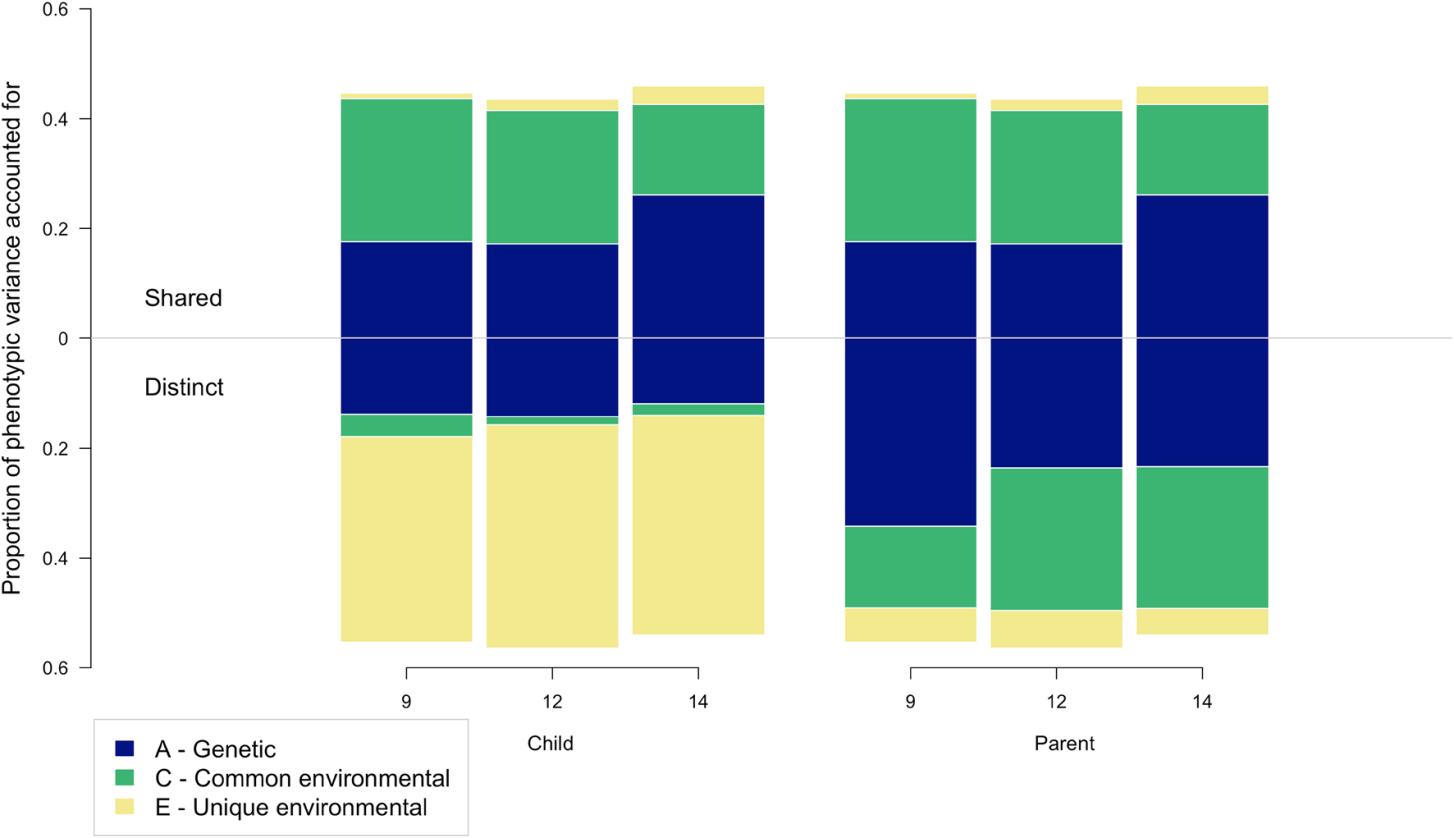
Proportion of variance in child- and parent-reported parenting explained by genetic, common environmental and unique environmental components of shared and distinct perceptions. *Note*: Proportion of shared variance plotted above *horizontal centre line*, variance distinct to each reporter (ordered child/parent) plotted below; *bars* divided based on the proportionate influence of genetic (*A*), common environmental (*C*) and unique environmental (*E*) factors; values from full psychometric model with scalar

The etiological results indicate that both genetic and common environmental factors were significant in explaining this shared variance at all ages (see Table 6, first three columns). Firstly, genetic factors from age 9 were a significant source of variance at all three measurement occasions (18 % at age 9; 6 % at age 12; 12 % at age 14). Similarly, age 9 common environmental influences also explained variance both at the occasion they were first measured (26 % at age 9) and subsequently (21 % at age 12; 10 % at age 14). Notably, there were differences in the extent to which new, age-specific genetic and common environmental factors emerged across development. While no new common environmental factors explained variance in shared perceptions significantly at age 12 or 14, genetic factors emerging at age 12 (11 % at age 12; 4 % at age 14) and age 14 (10 % at age 14) were significant. Unique environmental factors—which, when estimated for shared variance, do not include measurement error—were non-significant (or explained < 5 % variance) at all waves. For ease of interpretation, these results are represented visually in Fig. 3 (upper section).

#### Distinct Perceptions

Parameter estimates from the lower section of the model, which decomposes variance that is distinct to each reporter, are presented with 95 % confidence intervals in the remaining 6 columns of Table 6. The sum of values from *either* child-specific *or* parent-specific perceptions in the “Total” row for each age and corresponding values from shared perceptions is equal to unity (e.g., at age 12: 0.27 + 0.16 [shared] + 0.06 + 0.51 [child-specific] = 1; 0.27 + 0.16 [shared] + 0.13 + 0.44 [parent-specific] = 1). This shows that, as specified in the path diagram in Fig. 2, all of the phenotypic variance in child- and parent-reports respectively is explained by the combination of shared and distinct perceptions.

##### Child-Specific

Genetic factors were significant in influencing children’s distinct perceptions of the negative parenting they received, but only at age 9 (14 % variance explained). Age-specific genetic factors at 12 and 14 were of slightly smaller magnitude and thus did not reach significance. Limited stability in child-specific variance meant that no variance component in this section of the model explained any variation in parenting perceptions across measurement occasions. Furthermore, in contrast to the shared perceptions, common environmental influences were universally negligible for child-specific perceptions. Instead, age-specific unique environmental factors, which, in this section of the model, include measurement error, explained a substantial majority of the variance in child-specific perceptions of negative parenting (37 % at 9; 40 % at 12; and 39 % at 14).

##### Parent-Specific

Parents’ distinct perceptions of negative parenting showed significant genetic influence at all three measurement occasions. This genetic influence was present in two forms. Firstly, age-specific variance was explained by genetic factors at age 9 (34 %), age 12 (16 %) and age 14 (15 %) respectively. Secondly, similar to the pattern in shared perceptions, genetic factors that emerged early on continued to influence variance at later ages. This was evident both for age 9 genetic factors (8 % at age 12; 5 % at age 14) and age 12 genetic factors (3 % at age 14). In contrast with those of their children, environmental influences on parents’ distinct perceptions of their parenting were predominantly common, rather than unique (i.e., loading on C rather than E). Common environmental factors accounted for 15, 23 and 18 % of the age-specific variance at 9, 12 and 14, and were also significant contributors to stable variance. Age-specific unique environmental influences were smaller but still significant in this section of the model, accounting for 6 % at both age 9 and 12, and 5 % at age 14.

Both child- and parent-specific perceptions of parenting are represented in the lower section of Fig. 3.

## Discussion

Studies of parenting and the parent–child relationship often seek objectivity of measurement, using observational techniques that index behaviors and interactions in a standardized way (Smith 2011; e.g., Bradley et al. 1989). Despite many clear benefits to this approach, observational studies cannot unpick the relationship between children and parents’ subjective experiences and the way their respective behaviors shape their interactions. Numerous studies have demonstrated the functional importance of subjective experience and differential perception within the home environment (e.g., Ohannessian and De Los Reyes 2014; see De Los Reyes 2011 for a selective review). Genetically-informative designs can be used to investigate the etiological basis of the shared and individual realities of which the parenting relationship is composed (e.g., Feinberg et al. 2001). When applied longitudinally, these designs can help clarify the nature of what is stable and what changes across development, in terms of shared and distinct perceptions of parenting and the influences that underpin them.

The current study is the first to examine, longitudinally, the influence of genetic and environmental factors on the shared and distinct perceptions of parenting held by children and their parents. The results are informative as to the nature of the information actually captured by child- and parent-reports of negative parenting—and, in particular, how they relate across this transitional period of development. We were able to ascertain the extent to which parent and child reports overlapped or were distinct from one another, both phenotypically and etiologically. Furthermore, the results also revealed the degree of developmental stability or change with which these etiological factors operated to shape the subjective and shared experiences of parenting for children entering adolescence. Individually and collectively, these findings have a number of implications for developmental research and theory. The key findings from the study are summarized below, and these implications discussed accordingly.

### Phenotypic Patterns in Shared and Distinct Perceptions of Parenting

Variance in child- and parent- reports of negative parenting was primarily reporter-specific, with an average of 0.37 for cross-reporter phenotypic correlations This finding of comparatively low inter-reporter agreement is in line with typical findings of low correlations between child- and parent-report data (Achenbach et al. 1987; Bell et al. 2001; Reidler and Swenson 2012). Despite this, even the lowest levels of between-reporter agreement in our study accounted for around one-third of the total variance in the phenotype. As such the amount of variance available for decomposition into genetic, common environmental and unique environmental influences in both sections of the model was sufficiently large as to be interpretable.

### Genetic Influence on Perceptions of Parenting

Genetic factors significantly influenced shared perceptions of parenting. This finding, which shows that the parenting children receive is correlated with their genes, can be interpreted as reflecting evocative gene-environment correlation. This interpretation is supported by two facets of the study design. First, the current study is a child-based design, in which passive gene-environment correlation—the other main form of genetic confounding in the home environment—is not estimated as genetic influence (Neiderhiser et al. 2004). This is because passive gene-environment correlation, which relates to scenarios where the child’s genes are associated with their environments due to their parents providing both, does not create differences between twins of different zygosity (Avinun and Knafo 2014). Put simply, where parents’ genes influence their parenting, this is assumed to occur similarly for each child (and thus appears as “C” in child-based designs) unless evoked by the behavior of the child. Second, because this section of the models used in the current study represents variance *shared* by child- and parent-reports. Studies using only child-reports of parenting are typically unable to separate genetic influence mediated through children’s behavior from that mediated through their *perceptions* (Taber 2010). Although parents’ perceptual biases may be similarly influenced by their own genes, such an effect is estimated in the common environmental component of variance in child-based designs where one parent rates both twins (McAdams et al. 2013; Neiderhiser et al. 2004). This means that genetic influence on shared perceptions of parenting is indicative of child-driven effects on parenting that are corroborated by children and their parents. The presence of these effects has been demonstrated using a range other study designs (e.g., Cecil et al. 2012; Marceau et al. 2013; O’Connor et al. 1998; see Paschall and Mastergeorge 2015 for a review).

Genetic factors also significantly influenced distinct perceptions of parenting, for both children and parents. Typically, genetic influence in the reporter-specific part of the psychometric model is interpreted as valid, additional information about the phenotype (Bartels et al. 2007a). This is because neither bias nor measurement error can mimic the systematic effects of genetic contributions to variance in the models (Hoekstra et al. 2008). Nevertheless, genetic influence on distinct perceptions still requires careful interpretation. Although genetic factors in the lower part of the models necessarily differ between reporters (they would otherwise have appeared in the shared perceptions above), they pertain to the *child’s genes*. In the case of children’s distinct perceptions, this means that genetic influence is interpretable as a combination of perceptual (genes influencing children’s experience of parenting) and behavioral (genes influencing behaviors to which parents respond) effects. Since these are indistinguishable in this part of the model, significant genetic influences on children’s distinct perceptions at age 9 means that the perceptual mediation of genetic influence on parenting cannot be discounted in studies that use child-report data. Future work could seek to explore specific cognitive styles that may influence children’s experience of their environments during development.

In the current study, the observed genetic influence on *parents*’ distinct perceptions of parenting cannot be the result of parents’ genetically influenced perceptual biases, as the genetic components of these models index the effects of *child* genes. This is because the genetically informative relationship (either identical or non-identical twinship) upon which the decomposition of variance relies, exists in the child generation. Therefore, genetic influence on parents’ distinct perceptions could instead reflect an evocative effect of children’s heritable behaviors on parenting that the children themselves do not perceive. This interpretation is supported by evidence suggesting that parents may be more reliable reporters than children (e.g., Shelton et al. 1996). This would explain why parents’ perceptions account for additional “real” (i.e., pertaining to the target negative parenting behaviors) phenotypic variance: if the amount that could manifest in the shared part of the model was restricted by the limited reliability of children’s reports. Interestingly, while genetic influences at age 9 are greater for parents’ distinct perceptions than those for shared perceptions, this pattern is reversed by age 14. It is possibly that this trend reflects children’s developing insight into the evocative effects of their behavior on the parenting they receive. Such development would result in the pattern observed in the results: of genetic influence appearing increasingly in the shared, rather than parent-specific sections. However, the observed trend is slight and further work is needed to test this possibility empirically. For now, the most robust conclusion to be drawn from the finding of genetic influence in the parent-specific variance is that parents’ perceptions of their parenting seem to be substantially influenced by children’s behaviors in ways of which the children are unaware.

### Environmental Influences on Perceptions of Parenting

Common environmental factors were significant contributors to shared variance in parenting. In psychometric models of parenting, shared variance may be most informative about what has been termed the objective environment (i.e., aspects of the family environment that can be observed by outsiders). However, distinct variance may be just as informative about the functional environment (i.e., aspects of the family environment that are experienced subjectively by family members, and can thus have different functional consequences; Feinberg and Hetherington 2001). The contributions of common environmental factors in the top section of our models indicate that the objective environmental characteristics of negative parenting occur on a family-wide basis. Put simply, these represent parenting characteristics that are experienced similarly by twin siblings, but not in a manner that is associated with the genes that the children share. Whether or not these include *parents’* genetic factors cannot be ascertained in the current design, though research into the etiology of individual differences in parenting using parent-based designs suggests this to be the case (Klahr and Burt 2014). However, parenting may also be strongly influenced by environmental factors originating in an individual’s own experience of the parenting they received as children, or associated with wider cultural, societal or socio-economic influences (e.g., Bradley et al. 2001b).

Environmental influences on the distinct perceptions of parenting differed markedly and systematically between children and their parents. At each age, environmental contributions to child-specific variance were exclusively unique (i.e., differing between twins in a family), whereas environmental contributions to parent-specific variance were mainly common (i.e., family-wide). Primarily, this difference is likely a result of the fact that the same parent rated their own parenting of both twins; whereas the twins rated the parenting they received individually. However, within this context, it is also clear that parents report treating their children more similarly than the children themselves perceive. Previous studies have indicated the differential experience of parenting as an important predictor of developmental outcomes (e.g., Abar et al. 2015; Feinberg and Hetherington 2001; Maurizi et al. 2012; Reiss et al. 1995). The current study cannot inform as to the functional consequences of the disparate views evidently held by children and parents, though this would be an avenue for future research. However, it is noteworthy that the extent of the disparity seen in the current study would be exaggerated by any greater unreliability of children’s reports, which would inflate estimates of unique environmental influence in child-specific variance.

### Stability and Change in Etiological Influences on Perceptions of Parenting

The developmental patterns of the etiological contributions to shared and distinct perceptions of parenting shed further light on the nature of the phenotypic information that these measures capture. Overall, shared perceptions were predominantly stable across time. This indicates that those aspects of parenting that children and parents perceive similarly are also the most consistent across time, into adolescence. Developmental stability must therefore be considered as a key characteristic of the objective, family reality that shared perceptions are thought to index (Feinberg and Hetherington 2001; Feinberg et al. 2001). Common environmental influences were the largest contributor to stability in the shared perceptions of parenting. Notably, age 9 common environmental factors remained significant at age 12 and 14, suggesting that the same family-wide influences remain important across this transitional period of development. The interpretations of these common environmental influences on shared perceptions outlined above—genetically influenced parent characteristics, environmental influences from parents’ own upbringing and wider cultural influences—are all consistent with a picture of longitudinal stability rather than change (Bradley et al. 2001b; Costa and McCrae 1988, 1997). Furthermore, the declining influence of these common environmental influences over time is consistent with expectations about changes in an individual’s relationship with their home environment as autonomy and the importance of peer relationships increase early in adolescence (Steinberg and Silverberg 1986).

Genetic factors also contributed to stability in shared perceptions of parenting, with age 9 factors again enduring across this developmental period. However, unlike common environmental influences, which only contributed to stability, novel genetic factors were seen to emerge at both age 12 and age 14, each explaining roughly 10 % variance in child- and parent-reports of negative parenting. This implies that parenting, as it was co-perceived by children and parents, was influenced by children’s behavior in two ways: stably (potentially through trait-like behaviors) and age-specifically (via genetically-influenced behavioral change at different ages).

Children’s distinct perceptions of parenting were characterized by a pattern of age-specific change. While genetic influences in this section were only significant at age 9, the subsequent trend was towards an explanation of change rather than stability. New genetic factors appeared to show some involvement in driving change, but failed to reach significance at either age 12 or 14—whereas genetic contributions to stability were negligible. If, as outlined above, genetic influence on children’s distinct perceptions of parenting was partially mediated through their perceptual biases, this process occurred differentially at different ages. Overall though, the influence of genetic factors on children’s distinct perceptions was far outweighed by unique environmental factors, which were exclusively age-specific. Unique environmental influences in children’s distinct perceptions could have resulted from either their differential interpretation of parenting behaviors or simply by unsystematic measurement error. Both of these explanations are consistent with age-specific effects, and a combination of the two seems likely. To what extent age-specific, differential perception of parenting within a family is truly in operation is a relevant question worthy of future study, as it could further inform on a longstanding question in the field of developmental behavior genetics: why children from the same family actually develop so differently from one another (see Dunn and Plomin 1991; Plomin and Daniels 1987; Plomin 2011; Turkheimer and Waldron 2000).

Parents’ distinct perceptions were also largely age-specific, though both genetic and common environmental factors played a significant role in producing the modest amount stability that was evident. Primarily though, where children’s behavior evoked parenting responses that were distinctly perceived by parents (i.e., genetic influence on parents perceptions), this process was also developmentally dynamic. Common environmental influence in parents’ distinct perceptions could have been due to a rater-effect—an artifact of the fact that the same parent rated both twins (Ask et al. 2014). However, if this was the case, the time-specific nature of these influences shows that it was not an effect that operated consistently each time they reported. This means that parents’ bias towards reporting similar parenting of their twins did not extend to dictate the kind of parenting they reported (e.g., parenting reported as equitable was not consistently either more positive or negative at all measurement occasions). Age-specific, common environmental influences on parents’ views of their parenting suggests an element of their perception of the relationship that was more related to the child’s age than to either the stable characteristics of the parent themselves, or any behavior that was specific to their children as individuals. If these influences reflected parents’ genes influencing their parenting (in ways not corroborated by child-reports, otherwise this variance would go into the shared section of the model), this also differed depending on the measurement occasion.

### Limitations

The results of this study should be considered in the context of some assumptions and limitations. In particular, the usual assumptions of the twin model apply (Plomin et al. 2013; Rijsdijk and Sham 2002). The validity of these assumptions has been affirmed using a range of methodological approaches (e.g., Derks et al. 2006; Kendler et al. 1994; LoParo and Waldman 2014). Of the standard limitations of the twin model, the conflation of the unique environmental (E) component and occasion-specific measurement error bears specific mention here. This limitation reduces the interpretability of, in particular, the substantial age-specific “E” influences in the child-specific section of the models, as noted in the discussion of these results above.

If the parents of MZ twins were either more keen to highlight similarities in their parenting or less able to distinguish their children than parents of DZ twins, estimates of genetic influence on parent-specific variance would be inflated (Neale and Stevenson 1989). However, the potential for parent reports to contribute additional, specific information has been shown for a range of child behavioral phenotypes (e.g., Arseneault et al. 2003; Ask et al. 2014). Many of these behaviors have been shown either to be involved in evoking different parenting styles, or to be influenced by the same genetic factors as parenting (see McAdams et al. 2014 for a review). Accordingly, it seems likely that genetic influence on parents’ distinct perceptions of parenting can be interpreted in the context of gene-environment processes.

## Conclusion

Overall, the results of this study demonstrate the rich potential of using genetic data to investigate subjective perceptions of parenting in a developmental context. Objective measures of parenting remain an important source of information about the environmental context of child development. However, the dynamic complexity of the parenting relationship, along with the clear potential for children and parents to experience it differentially, necessitates taking a broader approach to its study. The influence of children’s genes on the parenting that they receive is a robust finding that emphasizes the dyadic nature of the parent–child relationship (Avinun and Knafo 2014; Kendler and Baker 2007). Our finding, that genetic factors influence not only the shared reality, but also (and in different ways) the individual realities of both children and parents, adds further nuance to this picture. Evidently, child-driven effects are complex enough to drive both stability and change in the parenting relationship, and to be both jointly or individually perceived. Our results also show that what is shared, in terms of children and parents’ perceptions of parenting, is more stable during the transition into adolescence than what is experienced subjectively. This raises the interesting possibility of differential perception as a contributing factor to the changes that occur in the parent–child relationship at this time. Future work investigating the functional consequences of age-specific discrepancies in perceptions of parenting could help to further elucidate the mechanisms—both genetic and environmental—by which parenting continues to relate to developmental outcomes in early adolescence and beyond.

## Footnotes

1 This contrasts with *parent*-*based* designs, such as the children-of-twin design, in which the genetically informative relationship is in the parent generation. The implications of this, in terms of interpreting the results, are covered in more detail in the discussion section.
